# Dual Role for FHY3 in Light Input to the Clock

**DOI:** 10.3389/fpls.2022.862387

**Published:** 2022-06-09

**Authors:** Bruce M. Rhodes, Hamad Siddiqui, Safina Khan, Paul F. Devlin

**Affiliations:** Department of Biological Sciences, Royal Holloway, University of London, Egham, United Kingdom

**Keywords:** circadian, clock, light, photoreceptor, transcriptome, mutant

## Abstract

The red-light regulated transcription factors FHY3 and FAR1 form a key point of light input to the plant circadian clock in positively regulating expression of genes within the central clock. However, the *fhy3* mutant shows an additional red light-specific disruption of rhythmicity which is inconsistent with this role. Here we demonstrate that only *fhy3* and not *far1* mutants show this red specific disruption of rhythmicity. We examined the differences in rhythmic transcriptome in red versus white light and reveal differences in patterns of rhythmicity among the central clock proteins suggestive of a change in emphasis within the central mechanism of the clock, changes which underlie the red specificity of the *fhy3* mutant. In particular, changes in enrichment of promoter elements were consistent with a key role for the HY5 transcription factor, a known integrator of the ratio of red to blue light in regulation of the clock. Examination of differences in the rhythmic transcriptome in the *fhy3* mutant in red light identified specific disruption of the CCA1-regulated *ELF3* and *LUX* central clock genes, while the CCA1 target TBS element, TGGGCC, was enriched among genes that became arrhythmic. Coupled with the known interaction of FHY3 but not FAR1 with CCA1 we propose that the red-specific circadian phenotype of *fhy3* may involve disruption of the previously demonstrated moderation of CCA1 activity by FHY3 rather than a disruption of its own transcriptional regulatory activity. Together, this evidence suggests a conditional redundancy between FHY3 and HY5 in the integration of red and blue light input to the clock in order to enable a plasticity in response to light and optimise plant adaptation. Furthermore, our evidence also suggests changes in CCA1 activity between red and white light transcriptomes. This, together with the documented interaction of HY5 with CCA1, leads us to propose a model whereby this integration of red and blue signals may at least partly occur *via* direct FHY3 and HY5 interaction with CCA1 leading to moderation of CCA1 activity.

## Introduction

The circadian clock is an internal regulator of biological processes that enhances fitness by ensuring living organisms are optimally in sync with the daily cycle of day and night (Creux and Harmer, 2019). A wide range of behaviours and metabolic reactions, environmental responses and even biotic interactions has been found to oscillate with a circadian rhythm that continues even under constant environmental conditions. In plants, circadian clock outputs control seedling establishment (Gommers and Monte, 2018), development (Henriques et al., 2018) and rhythms of leaf movement (Woodley Of Menie et al., 2019). Replenishment of the photosynthetic machinery is maximised around dawn (Dodd et al., 2015) while usage of starch reserves overnight is carefully timed to coincide with the duration of night (Graf et al., 2010). The clock also forms the basis of timekeeping for the measurement of day length in the regulation of flowering time, tuber formation and bud dormancy (Rodriguez-Falcon et al., 2006; Ibanez et al., 2010; Song et al., 2015) and, consequently, has a significant impact upon agriculture. Many plant responses also vary in magnitude at different times of day. Triggers received at certain times of day may be better indicators of environmental information than those received at other times. In such cases, the clock acts as a “gate” which closes at certain times, constraining these responses. For example, response to temperature is much greater around subjective dusk (Grundy et al., 2015). Light induction of gene expression is much more prominent during the subjective day (Millar and Kay, 1996; Fraser et al., 2021) and even responses to plant hormones is gated to be maximal at certain times of day (Covington and Harmer, 2007).

The circadian clock, itself, is acutely responsive to light. Light forms the most prominent indicator of dawn and dusk and so light, along with temperature, forms a key input to entrain the clock (Oakenfull and Davis, 2017). Although circadian clocks run with a precisely repeating period of approximately 24 h, a limited range of different period lengths can be observed within a population meaning that most individuals require minor adjustments to the phase of their clock to keep it in time with the diurnal cycle. Equally, latitudinal clines can be observed in circadian period length. Differences in period length within a species are thought to be an advantage in dealing with the varying stresses associated with different latitudes but this also requires an ability to reset the clock on a daily basis (Michael et al., 2003). Finally, as daylength changes through the year, minor adjustments are needed and the final pattern of the circadian cycle may be a combination of resetting signals at dawn and dusk that enable optimal adaptation to the different seasons (Hearn et al., 2018).

The photoreceptors mediating light input to the clock have been well characterised. Both red and blue wavelengths can adjust the clock, perceived by the red-absorbing phytochromes and blue-absorbing cryptochromes and Zeitlupe photoreceptor (Somers et al., 1998; Devlin and Kay, 2000; Yan et al., 2021). As an indication of how important light input is within the plant circadian system, the majority of the genes that make up the plant central clock have been demonstrated to be light regulated (Oakenfull and Davis, 2017). The plant clock, itself, consists of interlocked transcriptional feedback loops (Hsu and Harmer, 2014). Two morning-phased myb transcription factors, CCA1 and LHY, act to repress evening-phased genes, *ELF3*, *LUX*, and *ELF4*, which encode the constituents of an evening complex that, in turn acts to repress a series of pseudo response regulator genes. *PRR9*, *PRR7*, *PRR5*, and *TOC1* (*PRR1*). The PRR proteins peak in that order in a sequence ranging through the day, and act to complete the loop by repressing *CCA1* and *LHY* expression. The action of TOC1 in regulation of *CCA1* expression has been shown to involve interaction with the TPS transcription factor, CHE (Pruneda-Paz et al., 2009), which binds a TCP binding site (TBS), GGTCC (or GGACC). At the same time, positive acting factors including RVE8, another myb transcription factor related to CCA1 and LHY, act in response to light to activate expression of many of the central clock genes including the *PRR* genes, *PRR9*, *PRR5*, and *TOC1*, and the evening complex genes, *LUX* and *ELF4*. The *RVE8* gene also shows circadian regulation, with its expression peaking at dawn and, like *CCA1* and *LHY*, being repressed by the PRR proteins.

As well as driving oscillation of the central clock, the clock components are also responsible for output from the central clock, targeting a significant proportion of the transcriptome (Michael et al., 2008). Both the negative actions of CCA1 and LHY and the positive actions of RVE8 are mediated by their association with evening elements in the promoters of their target genes and the evening element has been found to be highly enriched in the promoters of rhythmic genes. CCA1 also binds to another TBS, GGCCCA (or TGGGCC) as well as GA (or CT) motif elements and potentially G-boxes (CACGTG) to mediate output from the clock (Kamioka et al., 2016).

We are also now beginning to understand more about how the mechanism of light input affects the genes of the central clock, itself. A number of signal transduction components acting downstream of the phytochromes and cryptochromes have been shown to act on central clock gene expression. The HY5 transcription factor acts downstream of both phytochromes and cryptochromes in light signalling. HY5 protein accumulates in response to light and associates with the ACE (ACGT) promoter element, which forms the core of the G-box, whereby it activates gene expression as part of a dimer with the related protein, HYH. HY5 associates with the promoters of most of the central clock genes but particularly acts in blue light input to the clock. The *hy5* mutant has a short period in blue light but not red or white light. It was recently demonstrated that HY5 levels are higher in blue light and considerably reduced in red light. Hence, HY5 confers information about the ratio of red and blue light (Hajdu et al., 2018). The light-signalling transcription factors, FHY3 and FAR1, also act a dimer to positively regulate expression of the central clock genes, *ELF4*, and *CCA1*, by binding to the FHY3/FAR1 binding site (fbs), CACGCGC, in the promoters of these target genes (Li et al., 2011; Liu et al., 2020). Consistent with this, loss of either FHY3 or FAR1 causes an almost complete loss of *ELF4* expression in white light. However, only a slight loss of the amplitude of *CCA1* and LHY expression was observed under the same conditions (Li et al., 2011), indicating that loss of either FHY3 or FAR1 does not stop the clock altogether in white light. However, the *fhy3* mutation does cause a much more dramatic effect in red light. In red light there is an almost complete loss of *CCA1* and *LHY* expression (Allen et al., 2006). Furthermore, analysis of the output gene, *CAB2*, also showed a wavelength-specific phenotype. *CAB2* expression in *fhy3* is arrhythmic in red light but shows limited rhythmicity in white light and completely normal circadian rhythmicity in blue light (Allen et al., 2006), indicating that action of FHY3 is also dependent on the proportions of red and blue light incident on the plant. Interestingly, FHY3 but not FAR1 protein directly interacts with HY5. Additionally, FHY3 interacts with a number of central clock proteins. FHY3 but not FAR1 also directly interacts with CCA1 and LHY in the regulation of *ELF4* expression, while both FHY3 and FAR1 directly interact with TOC1 and PIF5 in the regulation of *CCA1* expression.

Here we have further investigated the role of FHY3 and FAR1 in light input to the clock. We confirmed that the red-specific disruption of rhythmicity of the *fhy3* mutant extends to a wide range of clock outputs and to several central clock genes. However, the *far1* mutant showed normal rhythmicity in all light conditions. Given the requirement for both FHY3 and FAR1 dimer components for transcriptional activation of *ELF4* expression *via* the fbs, this suggested that the red-specific disruption of rhythmicity in *fhy3* likely reflects and additional role for FHY3, possibly related to its specific protein interactions. A microarray analysis comparing the circadian transcriptome in white light and red light revealed a series of coherent changes in the patterns of rhythmicity, which may explain the greater severity of the *fhy3* phenotype in red versus white light. Most notably, the proportion of the transcriptome showing rhythmicity was considerably reduced in red, while the amplitude of the expression of a number of clock genes was also reduced. Specifically, the morning-phased genes, *CCA1*, *RVE8*, and *PRR9*, and the evening phased genes, *LUX* and *ELF4*, damped low, while *PRR5* damped high. Simultaneously, the importance of the evening element and the G-box among rhythmic genes in red light was reduced. A general decrease in mean expression among rhythmic genes peaking during the night was also observed in red light, while genes showing reduced mean expression in red were also found to be strongly enriched in G-box elements. The importance of the G-box among genes showing differential expression patterns in white and red light, coupled with the known variation in HY5 levels in response to changes in the proportion of red and blue light, is consistent with a key role for HY5 in this transition between white and red light configurations. A similar investigation of the *fhy3* transcriptome revealed a loss of rhythmicity in the majority of output genes in red light. At the same time, almost all central clock genes showed a dramatic loss of amplitude, with *ELF3* and *LUX* becoming completely arrhythmic. In contrast, *PRR5*, uniquely, showed an increase in amplitude. Genes becoming arrhythmic in *fhy3* showed enrichment of the CCA1-targetted TBS site, TGGGCC, suggesting that CCA1 action is disrupted in *fhy3*. In contrast, the fbs element was only found to be enriched among genes showing a reduction in mean expression in *fhy3* and was not enriched among those losing rhythmicity, supporting the proposal that the red-specific arrhythmicity of *fhy3* is not simply related to the role of FHY3 as a transcription factor in directly activating clock genes. Rather, our findings point to the specific interaction of FHY3 but not FAR1 with CCA1 being a potential explanation for this phenotype. On that basis, we propose that FHY3 and HY5, respectively, may form a key mechanism for integration of red and blue light signals to the clock, with FHY3 acting in red and white light and HY5 in blue, possibly *via* their interaction as a complex with the CCA1 central clock protein.

## Materials and Methods

### Plant Materials and Growth Conditions

The *fhy3-*4, *far1*-2, and *fhy3*-4 *far1*-2 mutants of *Arabidopsis thaliana* and their isogenic wild type in the No-0 ecotype have been described previously, as have the *fhy3-*4, *far1*-2, and *fhy3*-4 *far1*-2 mutant and isogenic wild type luciferase reporter lines containing *CAB2:LUC* and *CAT3:LUC*, *TOC1:LUC* and *ELF4:LUC*, also in the No-0 ecotype (Wang and Deng, 2002; Li et al., 2011). In all experiments, seeds were sterilised in 30% bleach, 0.02% Triton X-100, sown on Murashige and Skoog (MS) medium containing 2% sucrose, then stratified for 3 days in darkness at 4 C. Following stratification, seeds were germinated and grown in 12 h white light/12 h dark cycles for 7 days prior to treatment conditions. White light for this consisted of equally mixed red light (λ-max 660 nm, 60 μmol m^–2^ s^–1^), blue light (λ-max 450 nm, 60 μmol m^–2^ s^–1^) provided by LEDs within Fytoscope FS80-RGBIR Minicabinets (Photon Systems International, Brno, Czechia). All experiments were carried out at 21 C.

Light conditions during luciferase bioluminescence imaging experiments were provided within the imaging chamber by a custom-made LED rig providing red light (λ-max 660 nm, 40 μmol m^–2^ s^–1^), blue light (λ-max 450 nm, 40 μmol m^–2^ s^–1^) or white light consisting of equally mixed red and blue light (total 40 μmol m^–2^ s^–1^).

For microarray analysis and for RT-qPCR analysis of *CCA1*, *LHY*, and *ELF4* rhythms, lighting conditions were provided by red light (λ-max 660 nm, 120 μmol m^–2^ s^–1^) LEDs within Fytoscope FS80-RGBIR Minicabinets (Photon Systems International, Brno, Czechia).

All light measurements were made using a StellarNet EPP2000-HR spectroradiometer.

### Luciferase Imaging

Luciferase imaging was carried out using a NightOwl ultra Cooled CCD (charge-coupled device) camera (Berthold Technologies, United Kingdom) as described by Siddiqui et al. (2016). Data were analysed by using Winlight image analysis software version 2.17 (Berthold Technologies, United Kingdom). Using this software, uniform, circular regions of interest were manually placed over the seedlings and quantification of bioluminescence counts from within these regions was automatically collected from the sequence of images taken and imported into a Microsoft Excel spreadsheet along with data for time of collection. Mean and standard error for bioluminescence for each seedling were then calculated. All data represent the findings of at least two independent experiments.

### Microarray Analysis

RNA extraction was carried out as described previously (Wang et al., 2011). Samples for our microarray analysis in red light in wild type and the *fhy3* mutant were taken at 24, 32, 40, and 48 h after the transfer to red light. Approximately 100 seedlings were collected for each sample. Our microarray hybridisation was carried out by the Nottingham Arabidopsis Stock Centre (Nottingham, United Kingdom) using Affymetrix ATH1 arrays. To enable the comparison of rhythmic gene expression in Arabidopsis in continuous white light, the publicly available Affymetrix ATH1 dataset, NCBI GSE8365 (Covington and Harmer, 2007) was selected. Following normalisation with our own microarray data, this was analysed for the identification of rhythmic gene expression alongside our microarray data using the methods developed here for this study. As in our assay, these seedlings in the NCBI GSE8365 dataset had been entrained in 12 h white light/12 h dark cycles for 7 days. These NCBI GSE8365 samples had been transferred to constant white light (120 μ mol m^–2^ s^–1^) and, after 24 h in constant light, 12 samples were harvested at 4-h intervals over the next 44 h. All data from both microarray experiments was normalised as a single dataset using the D-chip programme (Li and Wong, 2001). Probes lacking a corresponding AGI code in the Arabidopsis genome TAIR10 version of the Affymetrix ATH1 probe assignment were excluded prior to subsequent analyses as were probes corresponding to mitochondrial genes and chloroplastic genes. In order to exclude non-expressed genes, those probes which had a mean expression for every genotype/treatment considered that was below 5% of the mean expression value of all probes across all arrays were discounted.

### Rhythm Analysis

Rhythmicity was determined based on a combination of two criteria: correlation to a sine wave with a period of 24 h and a minimum change in expression from peak to trough. The correlation approach was based on that described by Devlin et al. (2003) for the analysis of co-expression patterns in microarray data. This was adapted for the analysis of rhythmic gene expression as follows. A Pearson correlation was performed to assess the degree of correlation to sine waves with period 24 h with mean and amplitude equal to 60 phased 1 h apart. The phase giving the highest *r* value for correlation was selected for each gene. A *t*-test for correlation (*r*) was then performed and genes showing a *p*-value of <0.01 were selected (*H*_0_: *r* = 0). Selected genes were then tested to determine whether they exceeded a minimum change in expression level from peak to trough. Genes showing either a predicted 1.5-fold change in expression from peak to trough or an absolute change in expression between minimum and maximum sample points of 250 times mean expression value of all probes across all arrays were selected. The predicted fold change in expression from peak to trough was calculated based on a comparison of the fold change between the nearest sample points to the calculated peak and trough and the predicted values at those same points for a sine wave with the same phase, the same mean expression level and a peak to trough variation of 1.5-fold. All calculations involved in selection were performed in Microsoft Excel and automated using visual basic.

### *Cis* Element Analysis

*Cis* element analysis was carried out using the MEME and DREME modules of the MEME Suite software package (version 4.12.0) (Bailey and Elkan, 1994; Bailey, 2011). The region 500 base pairs upstream of the transcription start site was used for analysis and MEME suite was run using the Cygwin interface https://www.cygwin.com on the Microsoft Windows operating system.

*Z*-scores for overrepresentation of specific phases among rhythmic genes containing recognised *cis* elements were calculated based on a rolling window of four phases. A bootstrapping approach was used to generate the background population for each phase window. A total of 100 random groups of rhythmic genes containing the same number of genes as were found within that phase window were selected from among all of the rhythmic genes in that same dataset. The number of occurrences of the element in question within the genes in the phase window was compared to the average from within the 100 random groups. All *z*-score calculations were performed in Microsoft Excel and automated using visual basic.

### Gene Ontology Analysis

Ontological analysis was performed using the PAGEMAN module of the MAPMAN software suite (Usadel et al., 2006), applying the defaults parameters. For data entry, genes within a selected group were given a score of 1 in place of the expected value for log2(expression change). *Z*-scores for over- or under-represented biological processes were then represented using the conditional formatting function in Microsoft Excel.

### RNA Extraction and qRT-PCR

RNA extraction and qRT-PCR were carried both out exactly as described previously (Wang et al., 2011). All gene expression values are expressed relative to an Arabidopsis *ACTIN2* housekeeping control. All data represent the findings of at least two independent experiments. The following primers were used for qRT-PCR: *CCA1*, TCGAAAGACGGGAAGTGGAA CG and GTCGATCTTCATTGGCCATCTCAG; *LHY*, AGTC TCCGAAGAGGGTCGTATAGC and TCACATTCTCTGCCAC TTGAGGAG; *ACTIN2*, TCCCTCAGCACATTCCAGCAGAT and AACGATTCCTGGACCTGCCTCATC.

## Results

### *fhy3* but Not *far1* Shows Specific Arrhythmicity in Constant Red Light for Multiple Non-direct Target Genes

**FIGURE 1:**
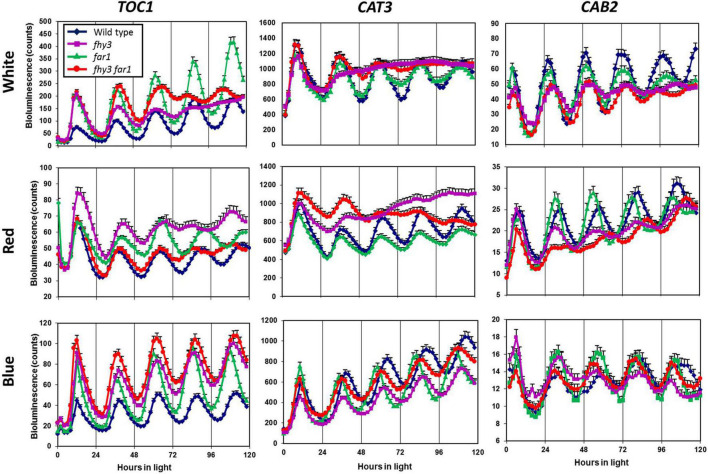
Loss of FHY3 but not FAR1 results in arrhythmic expression of non-target genes in the presence of red light. Wild type, *fhy3*, *far1*, and *fhy3 far1* mutant seedlings containing either the *TOC1:LUC*, *CAT3:LUC*, or *CAB2:LUC* transgenes were germinated and entrained in 12 h white light: 12 h dark cycles for 1 week before transfer to either constant white (red + blue), red or blue light. Luciferase bioluminescence was then recorded every 2 h. Data represent the means of at least 11 seedlings ±SE.

To examine the impact of both FHY3 and FAR1, individually and in conjunction, on the function of the circadian clock in various light conditions, we used luciferase reporter constructs to examine the circadian expression patterns of two clock output genes and one central clock gene in the *fhy3*, *far1*, and *fhy3 far1* double mutants in constant red, white and blue light. Seedlings were entrained to light dark cycles before release into constant light. The evening-phased central clock gene, *TOC1*, showed robust rhythmicity in wild type seedlings in red, white, and blue light. Similarly, *TOC1* expression was robustly rhythmic in all conditions in *far1* mutant seedlings. In contrast, although *fhy3* single mutant and *fhy3 far1* double mutant seedlings showed robust *TOC1* rhythmicity in constant blue light, both lines displayed a severe disruption of *TOC1* rhythmicity after two cycles of oscillation following release into constant white or red light (Figure 1). The evening-phased output gene, *CAT3*, and the morning-phased output gene, *CAB2*, behaved very similarly to *TOC1* in that circadian rhythmicity was dramatically disrupted in red or white light in the *fhy3* and *fhy3 far1* mutants but not in *far1* mutants (Figure 1). *CAT3* expression in *fhy3* and *fhy3 far1* was disrupted after only a single cycle in red or white light, ultimately resulting in an apparent arrhythmic phenotype in these mutant lines. None of *TOC1*, *CAT3*, and *CAB2* contain the fbs promoter element, meaning that these are not direct target genes for FHY3 and FAR1. These observations demonstrate that the disruption of rhythmicity of these non-target genes is specific to the absence of FHY3 only, and is not affected by absence of FAR1. Furthermore, the patterns of rhythmicity of these three genes confirm that the phenotype only occurs in the presence of red light in each case. These patterns reinforce the proposal that the previously observed red-light specific role for FHY3 in the circadian clock is distinct from its function as a positive acting transcription factor as part of a dimer with FAR1, where loss of either component has been shown to result in reduced function.

### Global Circadian Gene Expression Patterns Differ in Red Light

In order to further examine the nature of the red-light specific circadian defect in *fhy3* mutants, we carried out a microarray analysis of gene expression over a 24 h period in constant red light in wild type and *fhy3* mutant seedlings. Seedlings were entrained in 12 h/12 h white light/dark cycles for seven days before transfer to constant red light and tissue collection. RNA extraction was carried out at 24, 32, 40 and 48 h after transfer. Our data for *CAB:LUC* oscillation in red and white light (Figure 1) corroborated our previous observation that the loss of rhythmicity was more extreme in red light than in white light (Allen et al., 2006). Consequently, we also took the opportunity to compare the wild type transcriptome in red light to that previously published for seedlings in white light in otherwise identical conditions of entrainment and light intensity. Our aim here was to look for potential differences which could underlie the impact of light wavelength on the *fhy3* phenotype. For this, we examined the publicly available white light microarray dataset of Covington and Harmer (2007). After normalisation of both white light and all red light data as a single dataset and removal of data for ambiguous probes, non-nuclear genes and non-expressed genes, analysis of circadian gene expression patterns was performed using a method modified from Devlin et al. (2003). For this, the Pearson correlation coefficient was calculated for each gene to a series of sine waves of period 24 phased 1 h apart across one whole circadian cycle. The sine wave giving maximum correlation coefficient (*r*) for each gene was recorded. To ensure high stringency in light of the low sampling resolution for the red light data, only those genes for which the maximum correlation gave a *p*-value of less than 0.01 using a *t*-test for correlation coefficient (*H*_0_: *r* = 0) and met minimum extrapolated peak-to-trough expression change criteria (1.5-fold change or an absolute change of 250 times the mean expression of all genes on the array across all timepoints) were accepted as circadian. The phase of the sine wave giving the maximum correlation was then recorded as being the phase of expression for that gene. Applying these criteria to the white light data revealed 2,438 genes displaying a circadian rhythm (Supplementary Table 1). This represents 13% of expressed genes which agrees with previous analyses which range between 6 and 31% (Harmer et al., 2000; Edwards et al., 2006; Michael et al., 2008).

**FIGURE 2:**
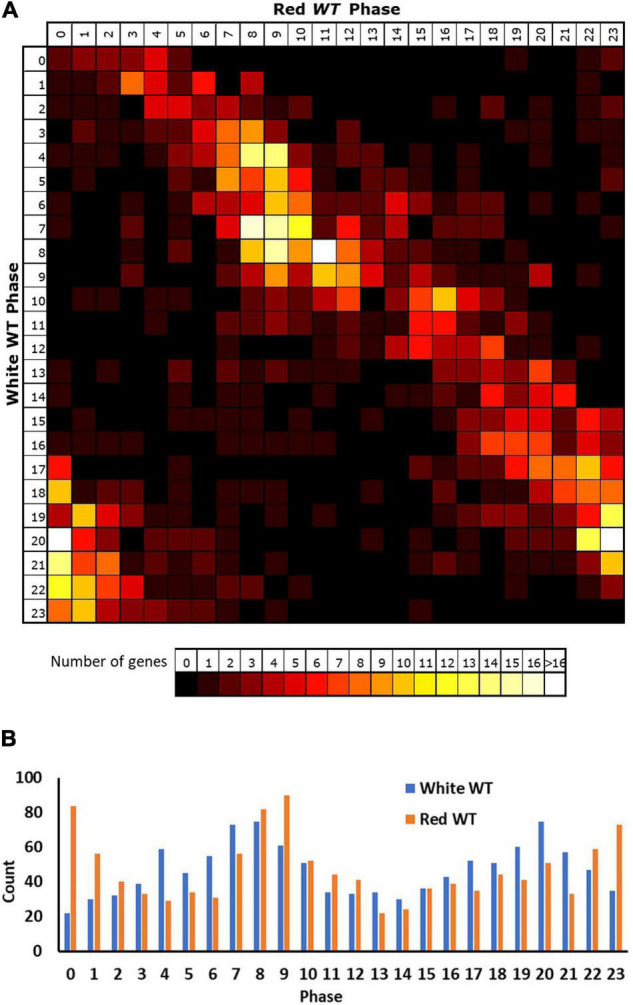
Rhythmic genes show a slightly delayed phase in red light. Phases of genes identified as rhythmic in wild type (WT) seedlings in both white and red light based on microarray data. **(A)** Genes were grouped by their calculated phases in each light condition and numbers of genes for each phase combination were represented as a heatmap. **(B)** Total number of genes displaying each phase in the two conditions.

Applying these criteria to our red light data revealed 5,915 genes displaying a circadian rhythm (31% of expressed genes, Supplementary Table 2). Analysis of the reasons for failure of genes to meet our selection criteria in either white or red light conditions indicate that lower numbers of genes in the white light data meet each of the two criteria (correlation and fold-change). A total of 79% of expressed genes in the white light dataset do not meet the expression change criteria compared to 60% of expressed genes in the red light dataset, consistent with the white light dataset collected by Covington and Harmer (2007) having a lower median dynamic range (median min–max range white = 1.26, red = 1.42; Supplementary Figure 1). At the same time, 69% of expressed genes in the white light dataset do not meet the correlation criterion, compared to 36% of expressed genes in the red light dataset. Despite the consistent 0.01 *p*-value cut-off used in both cases, it is probable that there would still be a reduced likelihood of a correlation for genes within the white light dataset given the fact that it covers two cycles rather than just one. Genes must, therefore, show good correlation with a sine wave over both cycles in the white light data to achieve the cut-off. The variable nature of the data means that there is likely to be a difference in the correlation of each cycle of actual data with a consistent sine wave applied across both cycles and the final correlation would represent a best fit “compromise” between the two cycles, reducing the *r* value that could be achieved by fitting a sine wave to a single cycle. We can, however, have a greater confidence in genes correlating in the white light data as a result. Given the latter, we considered the white light rhythmic transcriptome as our baseline for comparison between white and red light and looked at the way in which genes identified as rhythmic in white light changed their behaviour in red light. Primarily, it is notable that only 46% of the genes (1,129) identified as rhythmic in white light were also identified as rhythmic in red light suggesting considerable loss of rhythmicity in red light (Supplementary Table 3). However, a comparison of the phases of expression of genes that were identified as rhythmic in both conditions revealed that these genes showed a similar phase in both conditions; though, the vast majority were shifted slightly later by 2–3 h in red light (Figure 2A). Consistent with this, in both conditions, the most frequent times of peak expression for all rhythmic genes was also similar, with high numbers of rhythmic genes peaking during the afternoon or late night. Again, though, these maxima were on average 2–3 h later in red light than in white light (Figure 2B), suggesting that the absence of blue light results in a delay in phase; though, this could equally be caused by a lengthening of period, given the single cycle of data available for red light.

**FIGURE 3:**
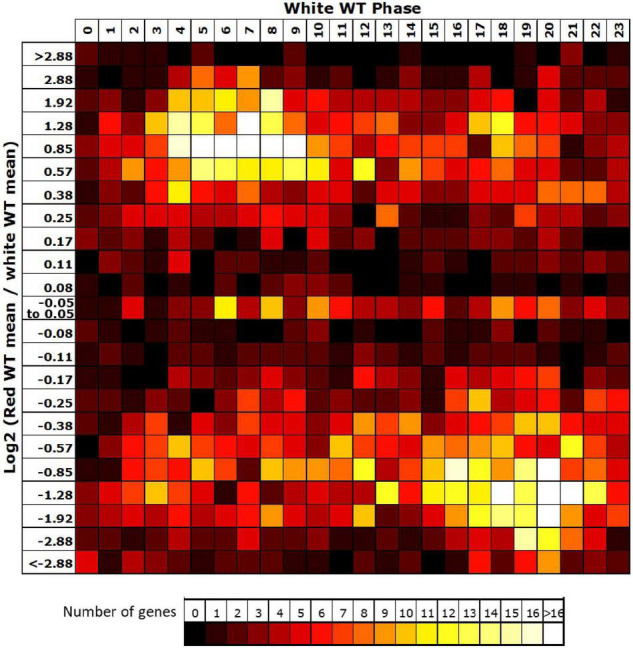
Night-phased genes in white light show a loss of mean expression in red. For all genes identified as rhythmic in wild type (WT) in white light, the change in mean expression in red light was plotted against calculated phase in white light. Bins for log2 change in mean expression also follow a log2 progression. Each expression change bin covers the range up to the value indicated, starting from the value of the preceding bin.

Given the loss of rhythmicity in red light observed for 54% of rhythmic genes in white light, we then investigated the reasons for failure to meet our criteria for rhythmicity among these genes. The most common reason behind loss of rhythmicity in red light was revealed to be a failure to meet our expression change criteria (67% of genes), suggesting that the absence of blue light resulted in a loss of amplitude. An analysis of the change in mean expression in red versus white light revealed that the majority of rhythmic genes in white light also showed a reduction in mean expression in the absence of blue despite the consistent light intensity in both conditions (Figure 3). Interestingly, the majority of these genes showing reduced expression in red were genes peaking during the subjective night (Figure 3), suggesting an important positive role for blue light at this time. The smaller group of genes showing an increase in mean expression in red showed a preference for genes peaking during the subjective day, the 180° phase difference, suggesting these may, indeed, include genes that are normally negatively regulated by those night time genes which now show lower expression in red.

We also observed differences in the enrichment of gene ontology terms that were associated with various biological processes among rhythmic genes in white versus red light. Rhythmic process terms enriched in white light included photosynthesis, major carbohydrate metabolism, amino acid synthesis, various aspects of secondary metabolism, as well as stress and redox signalling (Supplementary Figure 2). Photosynthesis and major carbohydrate degradation continued to be enriched among genes that were rhythmic in red light as did stress responses. However, minor carbohydrate metabolism (raffinose/trehalose), amino acid synthesis, and many secondary metabolic processes were no longer enriched. Instead, these processes were enriched among genes which became arrhythmic in red light (Supplementary Figure 2).

### Central Circadian Clock Gene Expression Patterns Differ Between White and Red Light

**FIGURE 4:**
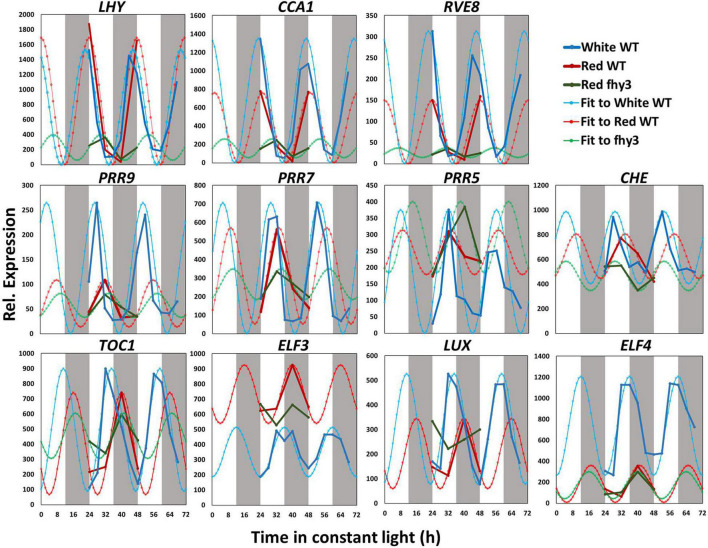
Changes in central clock gene expression in white light, red light and in the *fhy3* mutant. Mean microarray data values and mathematically fitted waveforms are shown for central clock genes in wild type (WT) seedlings in constant white and in wild type and *fhy3* seedlings in constant red light. Grey bars represent subjective day and night.

We then compared the expression patterns of the central clock genes in white light and red light-grown seedlings. The raw data and fitted sine waves are shown in Figure 4. All clock genes analysed continued to be rhythmic in red light and, consistent with global patterns of gene expression, most of the clock genes showed a slightly delayed phase in red (Figure 4 and Supplementary Table 4). However, more noticeable differences were observed in amplitude of expression of specific central clock genes in red. Amplitude was reduced in the morning-phased genes, *CCA1* and *RVE8*; in the daytime-phased genes, *PRR9*, *PRR5*, and *CHE*; and in the evening-phased genes, *LUX* and *ELF4* (Figure 4 and Supplementary Table 4). *ELF4*, particularly, showed a dramatic decrease in amplitude and mean expression. This very low *ELF4* expression in red was confirmed by analysis of bioluminescence in seedlings containing an *ELF4:LUC* transgene (Supplementary Figure 3). It is also notable that, while the amplitude reduction seen in *CCA1*, *PRR9*, *LUX*, and *ELF4* was a result of decreased peak expression, the reduced amplitude observed in *PRR5* was distinct in being the result of an increased trough value. The pattern of *ELF3* expression was also remarkable in showing a dramatic increase in mean expression level without showing any change in amplitude. In this comparison, both assays were carried out in the same light intensity suggesting that these changes in central clock gene expression are result of the specific absence in blue wavelengths and that this non-redundant nature of red and blue light input pathways to the clock may be the result of differential regulation of distinct groups target genes within the clock by the two pathways.

### Promoter Element Enrichment Differs Between the White and Red Light Circadiome

**FIGURE 5:**
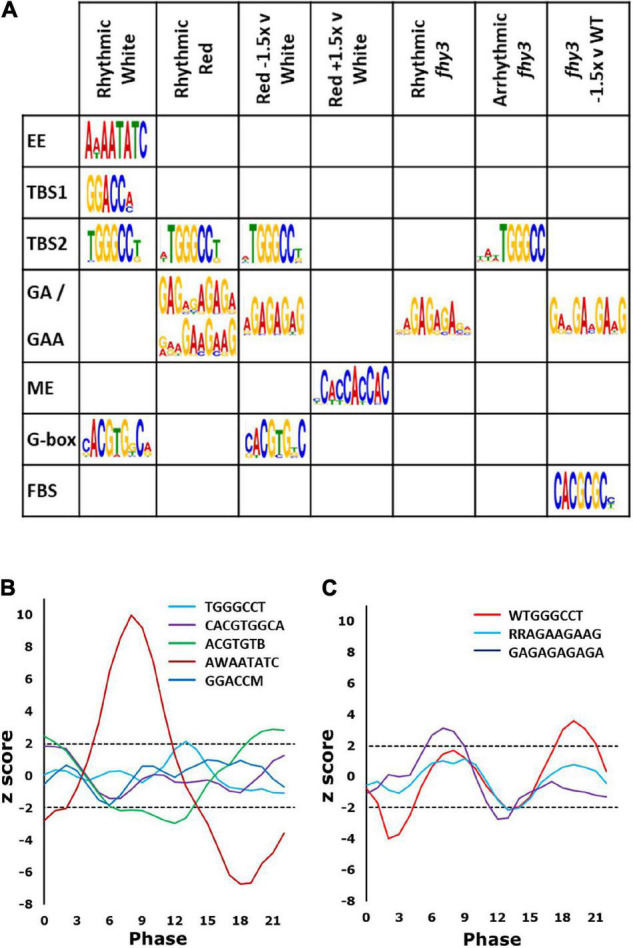
Promoter elements enriched in rhythmic genes. **(A)** Recognised elements enriched in genes in the categories indicated. Rhythmic White (rhythmic in wild type in white light); Rhythmic Red (rhythmic in wild type in red light); Red –1.5x v White (rhythmic genes in white light showing mean expression in red light ≤1.5-fold that in white light); Red +1.5 v White (rhythmic genes in white light showing mean expression in red light ≥1.5-fold that in white light); Rhythmic *fhy3* (genes which maintain rhythmicity in *fhy3* in red light); Arrhythmic *fhy3* (genes which lose rhythmicity in *fhy3* in red light); *fhy3* –1.5 v WT (genes with mean expression in *fhy3* in red light ≤1.5-fold that in wild type in red light). **(B)** Phase enrichment among Rhythmic White genes containing the elements indicated. Note two variants of the G-box are shown. **(C)** Phase enrichment among the Rhythmic Red genes containing elements indicated. Phase enrichment is represented by *z*-score and plotted as a three-point moving average. Dotted lines indicate *z*-scores corresponding to *p* = 0.05. *Z*-scores greater than 1.96 or less than –1.96 indicate significant enrichment for a given phase.

Promoter element enrichment analysis using DREME and MEME (Bailey and Elkan, 1994; Bailey, 2011) for the white light circadiome revealed several previously characterised circadian clock-associated *cis* elements within the region 500 bp upstream of the transcription start sites of rhythmic genes: the evening element (AAAATATC), bound by CCA1 and LHY (Harmer et al., 2000); two TCP binding sites (TBS): the core GGTCC (or GGACC) which is bound by CHE (Pruneda-Paz et al., 2009) and the GGCCCA (or TGGGCC) site, bound by CCA1 (Kamioka et al., 2016); and a G-box variant (CACGTG), associated with binding of HY5, PIFs and PILs (Toledo-Ortiz et al., 2003; Figure 5A).

Both TBS elements and the G-box showed significant enrichment, more specifically, in the region 50–100 bp upstream of the transcription start sites of rhythmic genes (Supplementary Figure 4). Analysis of the phase of genes containing each of these elements revealed that the genes possessing the evening element were significantly more likely to display a phase of peak expression in a window centred on phase 8 (afternoon), while genes containing a G-box were significantly enriched in a window centred around dawn (Figure 5B), consistent with previous observations (Michael et al., 2008). Those containing the TBS element, TGGGCC, were significantly enriched in a window centred on phase 14 (early night) (Figure 5B).

Promoter element enrichment analysis for the red light circadiome revealed only a partially overlapping set of enriched elements. The TBS element, TGGGCC, was again found; though, genes containing it showed a later peak of phase enrichment than was observed in white light, at around phase 19 (Figures 5A,C), consistent with the slightly later phase of expression of rhythmic genes in red. Curiously, neither the evening element nor the G-box were enriched in the red circadiome. Instead, two additional GA motifs were observed (GAGAGAGAGA and RRAGAAGAAG) (Figure 5A). Both are very similar to elements that are bound by CCA1 (Kamioka et al., 2016). Genes containing the GA element were also shown to be upregulated as a result of TOC1 over expression (Gendron et al., 2012). Genes possessing the GA motif were significantly more likely to display a phase of peak expression in a window centred on phase 8 (Figure 5C). Intriguingly, the disappearance of G-box enrichment in the red circadiome is accompanied by the observation that those genes which were rhythmic in white light and which showed a decrease in mean expression in red light (>1.5-fold decrease) did show an enrichment of the G-box (Figure 5A). The G-box is a target of HY5 which has been shown to bind to the promoters of a number of clock genes, with binding enhanced by blue light. It specifically regulates *PRR5*, *LUX*, and *ELF4* and has been predicted to regulate *CCA1* (Li et al., 2011; Hajdu et al., 2018), all of which show reduced amplitude in red. Finally, those genes showing an increase in mean expression in red light (>1.5-fold increase) showed a specific enrichment for presence of a morning element-related *cis* motif (Figure 5A), the morning element, enriched in the promoters of morning-phased genes (Harmer and Kay, 2005) which is consistent with the observation that the majority of genes showing upregulation in white versus red showed a morning phased expression (Figure 3).

In all, this suggests that the changes in patterns of circadian expression in the absence of blue light may be related to the role of HY5 in integrating the relative quantities of red and blue light. It is also notable that the evening element, which was specifically enriched in genes rhythmic in white light in many studies (Harmer et al., 2000; Michael et al., 2008), was not enriched among genes showing rhythmicity in red light. Along with the analysis of clock gene expression, this further indicates the difference in the relative importance of the central clock proteins responsible for rhythmicity, itself, for plants growing in the absence of blue light. This is, furthermore, consistent with our proposal that the relative importance of FHY3 may be greatly enhanced as a result of the loss of some redundancy in the clock system under the specific conditions created by the relative reduction in blue versus red light input.

### The *fhy3* Mutation Causes Dramatic Changes in the Circadian Transcriptome in Red Light

**FIGURE 6:**
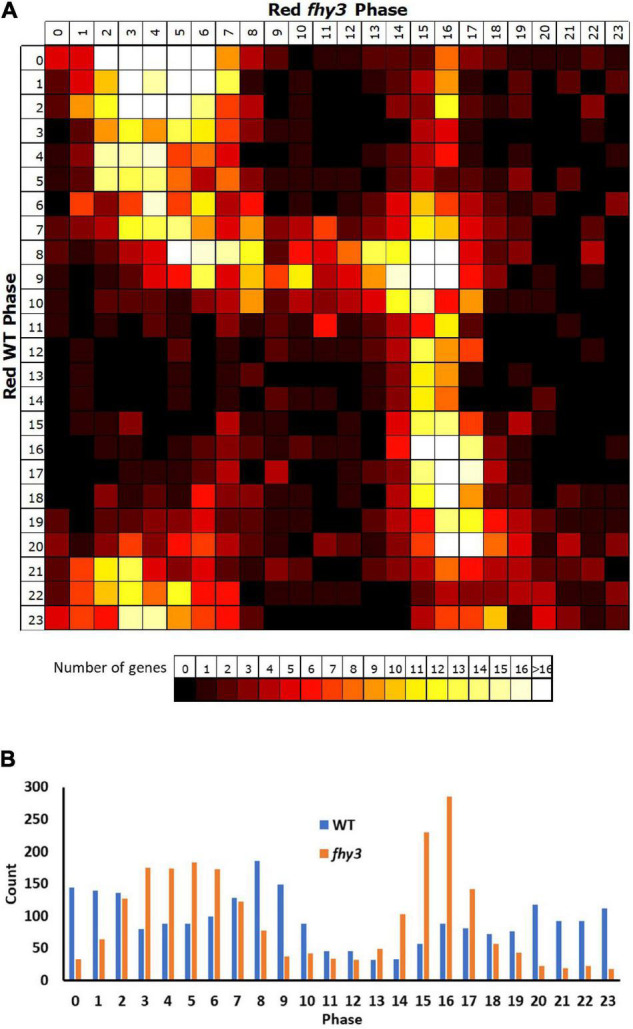
Rhythmic genes show a concentration in morning and early night phases in *fhy3* in red light. Phases of genes identified as rhythmic in both wild type and *fhy3* in red light based on microarray data. **(A)** Genes were grouped by their calculated phases in each genotype and numbers of genes for each phase combination were represented as a heatmap. **(B)** Total number of genes displaying each phase in the two genotypes.

Comparison of the circadiome of the wild type and *fhy3* mutant in red light revealed a dramatic reduction in the number of genes which remained rhythmic in *fhy3* (2,258 of the 5,915 rhythmic genes identified in wild type; Supplementary Table 5). Thus, although the majority of genes that were rhythmic in wild type lost rhythmicity, *fhy3* was not completely arrhythmic. Comparative analysis of the phases of peak expression of genes that remained rhythmic revealed an unexpected profile. Rather than a consistent phase relationship between wild type and *fhy3*, rhythmic genes in *fhy3* appear concentrated in one of two windows of peak phase, centred on phases 4 and 16 (4 h after subjective dawn and 16 h after subjective dawn), with the majority of genes that were phased at other times in wild type, shifting to one of these two phase windows in *fhy3* (Figure 6). Genes peaking around subjective dawn or phase 0 in wild type shifted slightly later to phase 4, genes peaking around late night in wild type shifting to phase 16 and genes peaking during the afternoon in wild type shifting to either phase 4 or phase 16 in approximately equal proportions (Supplementary Figure 5).

**FIGURE 7:**
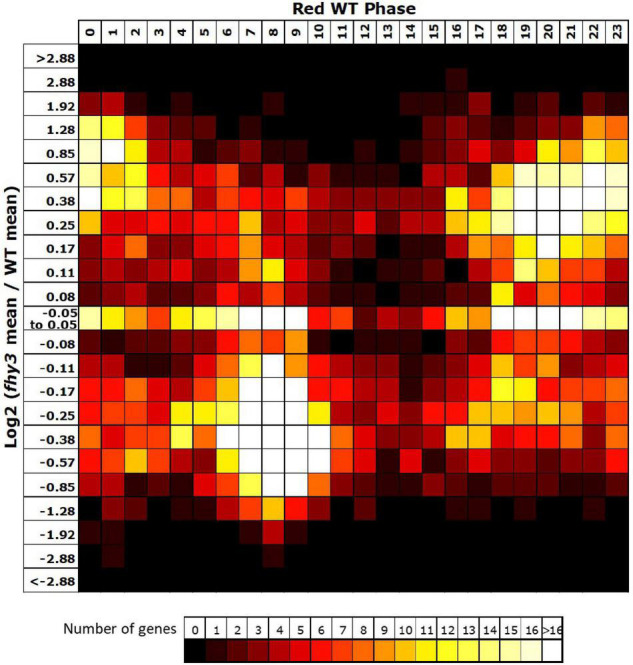
Afternoon-phased genes in wild type show a loss of mean expression in *fhy3* in red light. For all genes identified as rhythmic in wild type in red light, the change in mean expression in *fhy3* was plotted against calculated phase in wild type. Bins for log2 change in mean expression also follow a log2 progression. Each expression change bin covers the range up to the value indicated, starting from the value of the preceding bin.

The vast majority (3,465 of 3,657) of the genes which lost rhythmicity in *fhy3* failed our rhythmic selection criteria on the basis of fold change in expression, suggesting severe loss of amplitude among the majority of cycling genes was the primary cause of arrhythmicity. We also analysed the change in mean expression in *fhy3* in all genes that were rhythmic in wild type in red light. Curiously, though, we observed that approximately equal proportions of genes showed increased and decreased mean expression in *fhy3*, suggesting that this loss of rhythmicity in *fhy3* was not simply a general loss of positive regulation of gene expression due to the loss of a light signalling component. Furthermore, there was a marked association between phase of expression in wild type and the occurrence of either an increase or decrease in mean expression in *fhy3*. The majority of those genes which showed a decrease in mean expression in *fhy3* showed a daytime phase in wild type, whilst the majority of those genes which showed an increase in mean expression in *fhy3* showed a night time or dawn phase in wild type (Figure 7).

In terms of biological processes among genes that became arrhythmic in *fhy3* versus those that maintained rhythmicity, an ontology enrichment analysis suggested that photosynthetic processes, minor carbohydrate metabolism (raffinose/trehalose), and brassinosteroid metabolism became arrhythmic in *fhy3*. Cell wall proteins, gibberellic acid metabolism and stress responses maintained rhythmicity in wild type and *fhy3*, while major carbohydrate degradation, secondary metabolism, and auxin and abscisic acid metabolism, although not significantly enriched among all genes showing rhythmicity in red light (irrespective of white light), did also show rhythmicity in *fhy3* (Supplementary Figure 6).

### Analysis of Central Clock Gene Expression Indicates Fundamental Circadian Defects in *fhy3*

**FIGURE 8:**
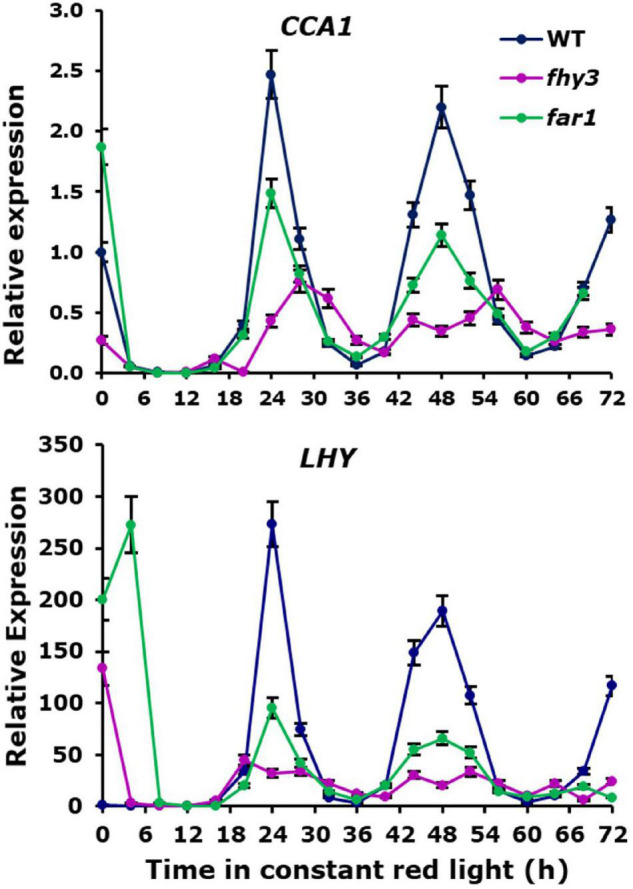
Confirmation of expression patterns of selected central clock genes in constant red light. Wild type, *fhy3* and *far1* mutant seedlings were germinated and entrained in 12 h white light: 12 h dark cycles for 1 week before transfer to constant red light. Expression of *CCA1* and *LHY* was calculated relative to Actin. Data represent the means of three biological replicates ±SE.

Analysis of the expression patterns of the central clock genes in *fhy3* in red light revealed the majority remained rhythmic by our definition; though, many showed a dramatic reduction in amplitude (Figure 4 and Supplementary Table 6). The evening genes, *ELF3* and *LUX*, however, experienced such a comprehensive loss of amplitude as to be classed as arrhythmic in *fhy3*. The dawn-phased clock genes, *CCA1*, *LHY*, and *RVE8*, all showed a dramatic loss of peak expression. *PRR9* and *PRR7*, showed both a loss of peak and gain of trough expression levels, damping to an intermediate mean expression level versus wild type levels in red. *PRR5* showed an increase in amplitude, courtesy of higher peak expression levels, whilst *CHE* showed a dramatic loss of peak expression similar to the morning-phased genes. Of the evening phased genes, *TOC1* damped high in *fhy3*, showing higher trough levels, consistent with our analysis of the effect of *fhy3* in red light in *TOC1:LUC* seedlings (Figure 1). In becoming arrhythmic, *LUX* also damped high in *fhy3*, whilst *ELF3* damped low. Curiously, *ELF4*, a direct target of FHY3 in its role as a transcription factor, showed little change in expression in *fhy3*. *ELF4*, however, was already damped to extremely low levels in the red light conditions used in this assay (Figure 4, Supplementary Figure 2, and Supplementary Table 6). This lack of impact on *ELF4* expression in red light, though, further supports the proposal that the red-specific loss of rhythmicity in *fhy3* is indicative of a mode of action of FHY3 that is distinct from its role as a transcription factor targeting *ELF4*. qPCR analysis in the same lines used for microarray analysis under identical conditions further confirmed the expression patterns of *CCA1* and *LHY* in wild type and *fhy3* seedlings in constant red light (Figure 8). Our qPCR analysis also included the *far1* mutant. In both cases the *far1* mutant showed only a slight reduction in amplitude in contrast to *fhy3*. It is also clear that the rhythmicity in the *far1* mutant is much more robust than that in *fhy3*. The loss of amplitude is consistent with FAR1 playing a role in the functioning of the FHY3-FAR1 dimer as a positive regulator of *CCA1* gene expression (Li et al., 2011) but the much more dramatic effects of the *fhy3* mutation are, again, consistent with an additional role of FHY3 in maintaining rhythmicity in these conditions.

Analysis of enriched *cis* elements associated with genes showing specific arrhythmicity in *fhy3* revealed only one recognised element, the TBS element, TGGGCC. In contrast, the other two elements that were associated with rhythmicity in red light in wild type seedlings, both variants of the GA motif, were the sole elements enriched in genes which maintained rhythmicity in *fhy3* (Figure 5 and Supplementary Figure 3). The absence of any enrichment of the fbs is also significant in that regard, further supporting the distinction from the mode of action of FHY3 as a transcription factor regulating gene expression *via* the fbs. Appropriately, an analysis of the *cis* elements enriched in genes that are rhythmic in wild type but which show a change in mean expression in *fhy3* revealed the enrichment of the fbs in genes showing at least 1.5-fold decrease in mean expression in *fhy3* (Figure 5 and Supplementary Figure 3). The TGGGCC motif is a CCA1 target (Kamioka et al., 2016). Together with the fact that CCA1 is a specific interactor with FHY3 but not FAR1 (Li et al., 2011), this supports our proposal that the red-specific defect in rhythmicity in *fhy3* may be a result of disrupted CCA1 transcriptional regulation activity. Indeed, we previously showed that FHY3 interaction normally represses the transcriptional activity of CCA1 (Li et al., 2011). Disrupted CCA1 action is also consistent with complete loss of rhythmicity in *fhy3* of two key CCA1 targets, *ELF3* and *LUX* (Figure 4).

## Discussion

### FHY3 Plays an Additional Role in Maintenance of Circadian Rhythmicity, Independent of FAR1

We previously demonstrated that the *fhy3* mutant showed a red light-specific arrhythmicity for several circadian clock-regulated processes. Growth in red or white light results in disrupted rhythmicity for leaf movement and for *CAB2* gene expression (Allen et al., 2006). Subsequent discoveries revealed FHY3 to be a transcription factor which dimerises with its close homologue, FAR1, to activate expression of genes possessing an fbs promoter element, including the *ELF4* central clock gene (Li et al., 2011; Liu et al., 2020). Loss of either FHY3 or FAR1 was shown to result in arrhythmic expression of *ELF4* in constant white light due to an extreme loss of *ELF4* expression (Li et al., 2011). However, the same study demonstrated that the wider loss of rhythmicity in white light, which was originally observed in the *fhy3* mutant (Allen et al., 2006), was only seen in the absence of FHY3 and not in the absence of FAR1 (Li et al., 2011). Here, we have expanded upon this by examining the *fhy3* and *far1* single mutants and the *fhy3 far1* double mutant in red, white and blue light. Both the *fhy3* mutant and the *fhy3 far1* double mutant displayed severely disrupted rhythmicity for expression of *CAT3*, *TOC1*, and *CAB2* expression in red and in white light but showed normal rhythmicity in blue. Conversely, *far1* mutants showed normal rhythmicity in red, blue, and white light, confirming that the role for FHY3 in maintaining rhythmicity in red or white light does not involve FAR1 and likely represents an additional mode of action for FHY3 beyond its role in the FHY3-FAR1 transactivating dimer. In this study we have gathered evidence from microarray analysis supporting a possible explanation for this phenomenon. One key distinction between FHY3 and FAR1 in their involvement in the circadian clock is their differential interaction with the central clock protein, CCA1 (Li et al., 2011). Only FHY3 but not FAR1 interacts with CCA1, and this interaction reduces the transcriptional repressive action of CCA1 (Li et al., 2011). We, therefore, speculated that the additional role for FHY3 in maintenance of circadian rhythmicity in red or white light may relate to this CCA1 interaction. In addition, a recent observation that HY5 acts as an integrator information on the ratio of red and blue light in the regulation of the clock offered a possible explanation for the occurrence of arrhythmicity in red and in white light but not in blue. HY5 protein is more stable in blue than in red and HY5 binds more strongly to the promoters of a number of central clock genes in blue than in red. Furthermore, *hy5* shows a mutant phenotype intermediate between that in red and blue light (Hajdu et al., 2018) and, thus HY5 action in wild type plants would create a potentially quite different circadian backdrop in these conditions against which FHY3 would be acting. HY5 is another interactor with CCA1 and, indeed, HY5 is another protein which interacts with FHY3 but not FAR1 (Li et al., 2011) suggesting all three may form a complex. Coupled with our earlier finding that FHY3 protein is stabilised in red light (Siddiqui et al., 2016), we, therefore, speculated that, perhaps FHY3 and HY5 together mediate a wavelength-specific regulatory effect on the clock which covers both red and blue input. Such an interaction would likely show a conditional redundancy whereby, in conditions with a high ratio of red to blue wavelengths (red or white light), FHY3 protein would be stable but HY5 protein would be destabilised. Conversely, in blue light, HY5 would be the dominant player. Thus, the impact of the loss of FHY3 would only be observed in red or white light. Consistent with this, our observations show that loss of FHY3 impacts clock gene expression much more in red and white light while Hajdu et al. (2018) have shown that the loss of HY5 much more strongly impacts on clock gene expression in blue light.

### Global Circadian Gene Expression Patterns Differ in Red Light

As an initial step in seeking support for these proposals, we sought to examine the impact of loss of FHY3 in red light on the wider transcriptome in the hope of better understanding this distinct FHY3 action. This analysis also afforded the opportunity to compare white light and red light circadian transcriptomes for the first time. We chose a correlation-based curve fitting approach for this as it forms a very convenient method to overall global comparisons of the whole circadian transcriptome, while still assigning circadian rhythmicity and phase to individual genes with statistical certainty. Such an approach also lends itself to analysis of low-resolution, short time course data. Our data indicated that approximately half of the genes that were observed to show circadian oscillation in constant white light showed a loss of rhythmicity in constant red light. In most cases this loss of rhythmicity resulted from a decrease in amplitude in red light. Genes which maintained rhythmicity in red light also preserved the same phase relationships as in white light; though, they showed a slightly later phase across the board. Our data also indicate a loss of mean expression level in red versus white light in many genes which peak during the subjective night. This implies that blue light may be particularly important at this time of day, possibly suggesting that it may be especially important in responding to light beyond the time of expected dusk or prior to the time of expected dawn as days lengthen toward summer. Central clock genes all maintained rhythmicity in red light; however, while many clock genes showed dramatic differences in amplitude in red versus white light, others maintained amplitude or even showed increased amplitude. Amplitude was reduced in *CCA1*, *RVE8*, *PRR9*, *CHE*, *LUX*, and *ELF4* as a result of loss of peak expression level. *ELF4*, particularly, showed a dramatic decrease in both amplitude and mean expression. These configurations point to a specific circadian role for blue light in determining the final patterns of expression of the central clock components observed in white light. The overall effect of the patterns of expression of the central clock components, however, does not appear to be related to simple loss of a positive effect of blue light input as light-induced genes, *LHY* and *TOC1* (Martinez-Garcia et al., 2000; Hsu et al., 2013) are among those that showed no loss of amplitude. A loss of amplitude in the light-responsive *PRR5* also belies this explanation as this is the result of an increase in trough levels, while the light-responsive *ELF3* shows no change in amplitude but dramatically increased mean expression.

Of the daytime and evening-phased clock genes, the triplet of *PRR5*, *LUX*, and *ELF4*, which showed a reduction in amplitude, have all been shown to be regulated by HY5 *via* differential binding of HY5 to the G-box in red versus blue light (Hajdu et al., 2018). The loss of the G-box among elements enriched among rhythmic genes in red light also points to HY5 potentially being a key factor behind the change in expression patterns among central clock genes in red versus white light. Furthermore, the simultaneous enrichment of the G-box among genes showing a decrease in mean expression level in red light also supports this proposal. This is consistent with the observation that HY5 input to the clock is known to change with the ratio of red to blue light. HY5 is stabilised and provides a strong input to the clock in blue, less so in white and less so still in red (Hajdu et al., 2018). On top of this, genes showing the greatest loss of mean expression in red versus white light in our study showed a strong tendency to peak between phase 18 and 21, which coincides with the exact range of peak expression observed by Hajdu et al. (2018) for *HY5* expression. At the same time, the CCA1 target element, the GA motif, became statistically enriched among genes found to be rhythmic in red light, while, the evening element, another CCA1 target element, became less important among rhythmic genes in red light, perhaps implying that changes in CCA1 activity may be an important difference between red and white light. Interestingly, the HY5 protein is also a specific interactor of CCA1 and so it is possible that differences in HY5 stability in white versus red may possibly also have an impact on CCA1 activity to alter the balance of clock coordination in red versus white light.

Our findings also suggest that there are some key differences in overt rhythms in white versus red. While rhythmicity within primary metabolism and stress responses appear to remain unchanged in white versus red light, there appears to be a loss of rhythmicity in raffinose and trehalose metabolism, amino acid synthesis and several secondary metabolic processes in red light. The impact of light wavelength on levels of secondary metabolism is well established and is an important aspect of the design of artificial lighting for horticulture (Darko et al., 2014) but the suggestion that that levels of blue light are also important in maintaining normal circadian regulation of such secondary metabolic processes and may form an additional factor for consideration by growers.

These findings represent the first comparison of the circadian transcriptomes in red and white light and, overall, reveal subtle differences in overt rhythms but, more importantly, a switch in emphasis within the central mechanism of the clock in red versus white light, although the maintenance of rhythmicity in all clock genes suggests that the components of the mechanism remain the same even if their relative importance or activity changes.

### Global Patterns in *fhy3* Indicate Potential Point of Action for FHY3

Our primary aim in examining global gene expression patterns in red light, however, was to examine the impact of the *fhy3* mutation on the functioning of the circadian clock. A comparison of rhythmicity in wild type versus *fhy3* revealed a dramatic decrease in the proportion of rhythmic genes in red light consistent with our earlier analysis of individual output genes. The importance of FHY3 in maintaining rhythmicity in red light is also emphasised by the fact that photosynthesis related processes are no longer overrepresented among rhythmic genes in *fhy3*. Of particular interest, though, was the impact of the mutation on the genes of the central clock. The majority of the clock genes maintained rhythmicity in *fhy3* in red light, including the two well-documented direct target genes of the FHY3/FAR1 transcriptional activating complex, *CCA1* and *ELF4*. *CCA1* did show a reduction in amplitude as has been observed previously (Liu et al., 2020); however, surprisingly, the evening complex gene, *ELF4*, showed no loss of amplitude in *fhy3* in these red light conditions, contrary to the dramatic loss of *ELF4* amplitude observed in *fhy3* in white light (Li et al., 2011). As noted above, *ELF4* expression is already extremely reduced in red light compared to white light in wild type seedlings and it is possible that the lack of impact of the *fhy3* mutation on *ELF4* in red is a result of *ELF4* expression already being effectively minimal. In contrast, there was a much greater impact of *fhy3* elsewhere in the central clock. The two other evening complex genes, *ELF3* and *LUX*, become arrhythmic in *fhy3*, with *ELF3* damping low level and *LUX* damping high. This points to the mis-regulation of these genes perhaps being key to the severe disruption of overt rhythmicity in the *fhy3* mutant. These genes are direct targets of CCA1, supporting our proposal that the interaction of FHY3 with CCA1 and the modulation of CCA1 transcriptional regulator function by FHY3 may be the key to the phenotype. Analysis of the *cis* elements enriched among genes that lose rhythmicity in the *fhy3* mutant, also supports this proposal. Genes becoming arrhythmic show enrichment of the TBS element, TGGGCC, bound by CCA1 (Kamioka et al., 2016). It may also be that the loss of this moderation of CCA1 activity also contributes to the gating defect in *fhy3*. *CCA1* expression is strongly promoted by light. It may be that FHY3 protein which is, itself, stabilised in light, is essential to shepherd the clock through the day, preventing excessive clock resetting by light.

The loss of *LUX* and *ELF3* rhythmicity and the already low levels of *ELF4* expression suggest that the evening complex would be likely to show an overall inability to fulfil its normal function as a cog in the clock in *fhy3*. Mutations in *LUX* and in *ELF3* have both been shown to result in extremely low *CCA1* and *LHY* expression, while *TOC1* expression damps high in *lux* and *elf3* mutants (Hazen et al., 2005; Dixon et al., 2011) exactly as was observed in *fhy3* in red. Again, *cis* element analysis is consistent with clock gene expression, with genes remaining rhythmic in *fhy3* showing enrichment of the GA motif associated with high TOC1 levels (Gendron et al., 2012). Similarly, loss of *ELF3* rhythmicity in *fhy3* in red light could also help to explain the previously observed loss of gating of light input to the clock in the *fhy3* mutant (Allen et al., 2006). The *elf3* mutant also shows a loss of gating of light input (McWatters et al., 2000; Covington et al., 2001).

Further evidence that the circadian defect in the *fhy3* mutant does not relate to its action as a transcription factor in the FHY3-FAR1 transcriptional activation complex comes from the fact that the target fbs *cis* element was not found to be enriched among genes becoming arrhythmic in *fhy3*. However, it was found among genes showing a loss of mean expression in *fhy3*. Thus, this second role of FHY3 is also disrupted in the mutant as would be expected but it appears to result in a more general decrease in target gene expression. Nevertheless, *CCA1* is one of the target genes of the FHY3-FAR1 transcriptional activation complex *via* the fbs element in its promoter and this may also contribute to the low levels of *CCA1* observed in *fhy3*. It is also of interest that, among rhythmic genes, there was a particular grouping of genes with peak phases 7–9 that showed a loss of mean expression in *fhy3*, suggesting that this may be the time at which the FHY3-FAR1 transcriptional activation complex normally acts most strongly. Consistent with this, FHY3 protein is strongly daytime expressed in light dark cycles (Li et al., 2011).

In addition to changes in amplitude among the central clock genes, the morning genes, *CCA1*, *LHY*, and *RVE*, along with the daytime gene, *PRR5*, all show a considerable delay in phase of between 5 and 7 h compared to wild type in *fhy3* in red light (Supplementary Table 6). The change in phase in genes which remain rhythmic in *fhy3* is reminiscent of the *cca1 lhy* double mutant phenotype which also shows a change in phase in constant light (Alabadi et al., 2002). The *cca1 lhy* double mutant assumes an earlier phase rather than a later phase (Alabadi et al., 2002); however, this provides a precedent for the fact that dramatically altered levels of all of the central clock components can cause a change in phase angle versus the preceding entraining stimuli. The fact that not all central clock genes in *fhy3* show this phase delay, indicates that there is also a change in the phase angle between the components of the clock loop. It is possible that this change in the relative phases of the clock genes may underlie the strong grouping among those clock output genes which remain rhythmic in *fhy3* into to phase groups, centred on phases 4 and 16. Output pathways from the clock are formed by the direct action of the transcription factors that make up the central clock loop and many output genes are targetted by more than one clock component due to combinations of *cis* elements in their promoters, with the ultimate phase of the target genes being regulated by additive effects. In this way a wide range of phases for output genes can be conferred by a relatively small number of central clock genes. Changes in the relative levels as well as the relative phases of the central clock gene expression may together cause a narrowing of the range of phases possible. In addition, it is possible that the differences in relative phase between the central clock components and also phase grouping among the output genes could, at least partly, be a result of the gating defect in *fhy3*, whereby improperly regulated light input causes clock resetting at inappropriate times of day. Indeed the strong shift in phase seen in *fhy3* among genes peaking in the subjective afternoon in wild type is consistent with the timing of the defect in gating previously observed in *fhy3* (Allen et al., 2006).

### HY5 and FHY3 Offer a Potential Mechanism for Plasticity in Red and Blue Light Input to the Clock

**FIGURE 9:**
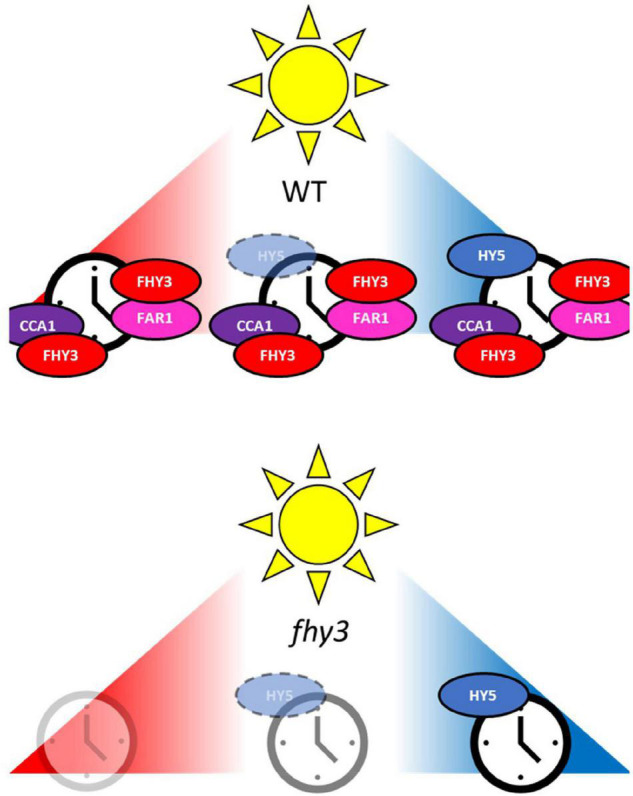
Proposed model for the integration of red and blue light into to the plant clock *via* FHY3 and HY5. FHY3 plays a dual role in light input to the clock. Firstly, FHY3 acts in conjunction with FAR1 to form a transactivating complex targeting the fbs element in the promoter of *CCA1* and *ELF4* genes and is required for high amplitude of their expression (Li et al., 2011; Liu et al., 2020). Secondly, FHY3 but not FAR1 is required for the maintenance of wider overt rhythms in constant white and, particularly, red light, where loss of FHY3 causes severely disrupted rhythmicity. Our evidence suggests that this involves moderation of CCA1 activity. FHY3 but not FAR1 interacts with CCA1 and moderates it transcriptional activity in the clock (Li et al., 2011) and we show here that loss of FHY3 particularly disrupts CCA1 target genes. Our findings further suggest that the wavelength specificity of the *fhy3* mutant phenotype in the maintenance of wider overt rhythms in constant light may reflect a conditional redundancy between FHY3 and HY5 in light input to the clock. We show here that genes most severely impacted in red light compared to white light are HY5 target genes. Levels and activity of HY5 decrease with decreasing ratio of blue to red light resulting in blue light-specific HY5 activity in light input to the clock (Hajdu et al., 2018). We propose that, in the absence of FHY3, HY5 compensates for FHY3 in maintaining rhythmicity in constant blue light. However, we propose that decreasing HY5 activity means that rhythmicity is disrupted in the *fhy3* mutant in constant white and severely disrupted in constant red light.

Our findings point to a conditional redundancy in the roles of HY5 and FHY3 in light input to the clock. Given the fact that HY5 protein shows enhanced stability in blue light while FHY3 protein shows enhanced stability in white and red light (Li et al., 2011; Siddiqui et al., 2016; Hajdu et al., 2018), this may offer a mechanism for plasticity in recruitment of photoreceptor input for the maintenance of normal rhythmicity. The complementary phenotypes of the *fhy3* and *hy5* mutants are consistent with such an arrangement. The *hy5* mutant shows a blue-specific phenotype, while *fhy3* shows a white- and red-specific phenotype. It is possible that this wavelength specificity is the result of conditional redundancy with FHY3 compensating for HY5 in red and white light and HY5 compensating for FHY3 in blue (Figure 9). However, although the stability of each is enhanced by specific wavelengths, both proteins none-the-less remain present in all light conditions so this conditional redundancy may depend on levels of the compensating protein falling below a certain threshold in each case. The fact that the *fhy3* mutant phenotype is more extreme than that of *hy5*, would then require that this threshold level for the two proteins is asymmetric. It is also likely that there would be some overlap in action of the two proteins in white light as, rather than showing a simple blue regulated stability, HY5 proteins levels actually respond to the ratio of red to blue light. Indeed, the fact that the *fhy3* phenotype is more extreme in red light than white light (Figure 1; Allen et al., 2006) is consistent with a limited amount of HY5 action in this proposed role in white. The fact that FHY3 but not FAR1 interacts with both CCA1 and HY5 leads us to further suggest that this integration may happen as a part of a three-way complex. Our evidence suggests that FHY3 action appears to be *via* moderation of CCA1 activity, something previously demonstrated at a molecular level (Li et al., 2011). However, we also saw some evidence of a change in CCA1 activity in white versus red light alongside evidence of changes in HY5 activity. It is, therefore, tempting to speculate that one point of convergence of red and blue light signals in input to the clock *via* FHY3 and HY5 may be in the moderation of CCA1 action.

## Conclusion

We have demonstrated that the phytochrome signalling component, FHY3, plays a second role in regulation of the circadian clock that is in addition to its previously described activity within and FHY3-FAR1 dimer as a transcriptional activator of *ELF4* and *CCA1* expression. Loss of only FHY3 and not FAR1 resulted in a red and white light-specific defect in rhythmicity. Our comparison of the transcriptomes of plants grown in red versus white light revealed key differences consistent with the proposal that the previously established role of HY5 as an integrator of the ratio of red to blue light may account for the wavelength specificity of the *fhy3* phenotype. HY5 and FHY3, therefore, appear to redundantly act to integrate red and blue light input to the clock. Such a plasticity in recruitment of photoreceptor signalling components in input to the clock would allow plants to adapt to a range of light environments. Furthermore, our analysis of the transcriptomes of wild type and *fhy3* mutant seedlings in red light provided strong evidence that FHY3 acts on central clock genes targetted by CCA1, in particular, *ELF3* and *LUX* to maintain rhythmicity. Based on the facts that both HY5 and FHY3 are known interactors with each other and with CCA1, and both moderate the transcriptional activity of CCA1, we propose that the two light signalling proteins, together, may, at least partly carry out this integration of red and blue light input *via* the modulation of CCA1 activity.

## Data Availability Statement

The data presented in the study are deposited in the Gene Expression Omnibus (GEO) repository, accession number GSE201929.

## Author Contributions

BR, HS, and PD contributed to project design. HS and SK prepared RNA for microarray analysis, carried out the bioluminescence analyses of the LUC reporter lines and the qRT-PCR assays. BR analysed the microarray data. BR and PD wrote the manuscript. All authors discussed the results and commented on the manuscript.

## Conflict of Interest

The authors declare that the research was conducted in the absence of any commercial or financial relationships that could be construed as a potential conflict of interest.

## Publisher’s Note

All claims expressed in this article are solely those of the authors and do not necessarily represent those of their affiliated organizations, or those of the publisher, the editors and the reviewers. Any product that may be evaluated in this article, or claim that may be made by its manufacturer, is not guaranteed or endorsed by the publisher.
