# Supplementary material for: Dual Role for FHY3 in Light Input to the Clock
Source: Front Plant Sci. 2022 Jun 9;13:862387. doi: 10.3389/fpls.2022.862387 (PMC9218818; doi:10.3389/fpls.2022.862387)
Supplement: Supplementary file 1 [file Data_Sheet_1.docx]

**Supplementary Figures**

**
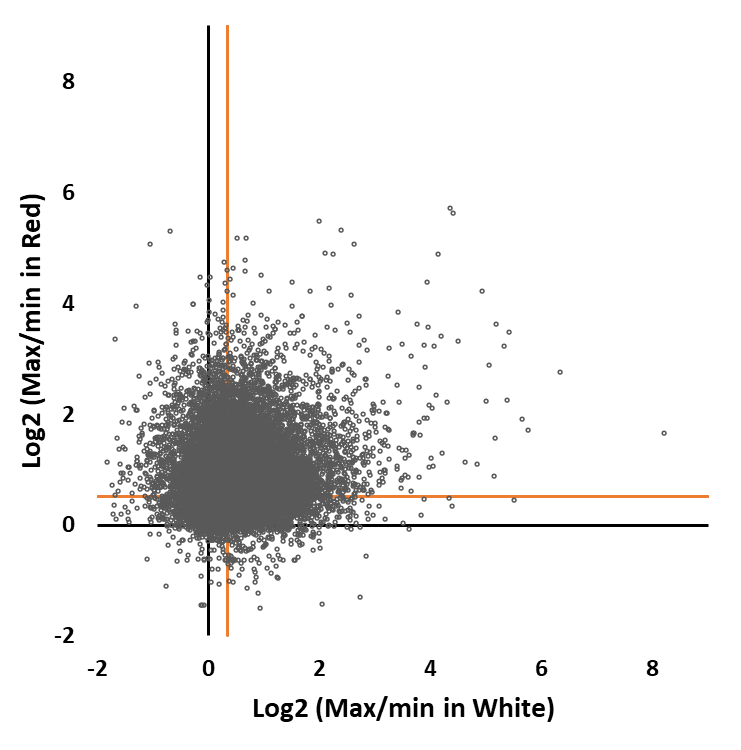
**

**Fig. S1. Genes shows greater median range of expression in the red light microarray dataset.** Range of expression expressed as Log2(Max/min) was calculated for each expressed nuclear gene across all time points in each of the two experiments (seedlings maintained in either white or red light). Orange lines represent the median range the two datasets.

**Fig. S2. Rhythmicity of gene expression within specific biological processes is differentially affected by light quality.** Overrepresented (green) and underrepresented (red) biological process categories associated with rhythmic genes in white light versus red light microarray data were calculated using the PageMan application. “Rhythmic WL” contains all genes rhythmic in white light; “Rhythmic WL & RL” contains genes that were rhythmic in white light and red light; while “Rhythmic WL only” contains genes that were rhythmic in white light but which became arrhythmic in red light. For clarity, categories are labelled according to parent ontology terms, though enrichment patterns of all child terms are represented for each.


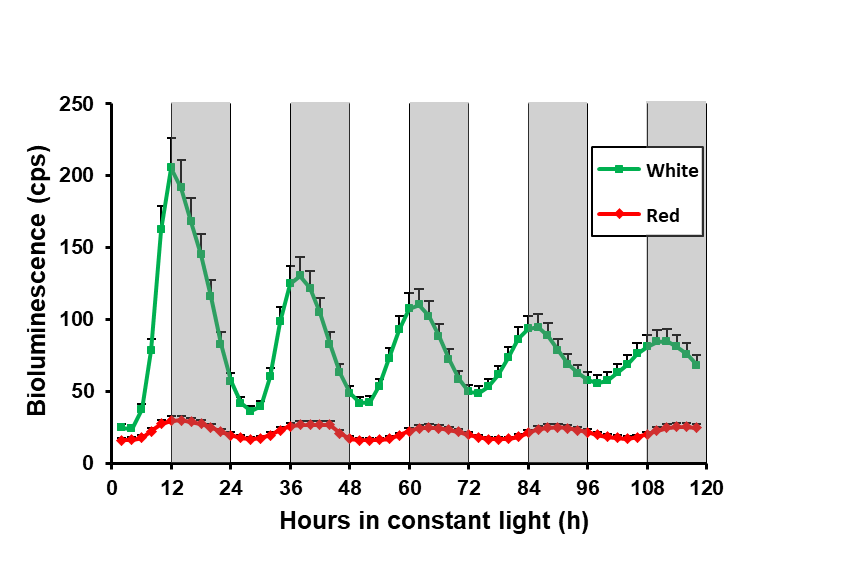


**Fig. S3. *ELF4::LUC* shows dramatically reduced expression red light.** Wild type seedlings containing either the *ELF4::LUC* transgene were germinated and entrained in 12 h white light : 12 h dark cycles for 1 week before transfer to either constant white (red + blue), red or blue light of the same total intensity. Luciferase bioluminescence was then recorded every two hours. Data represent the means of at least 18 seedlings +SE.

**
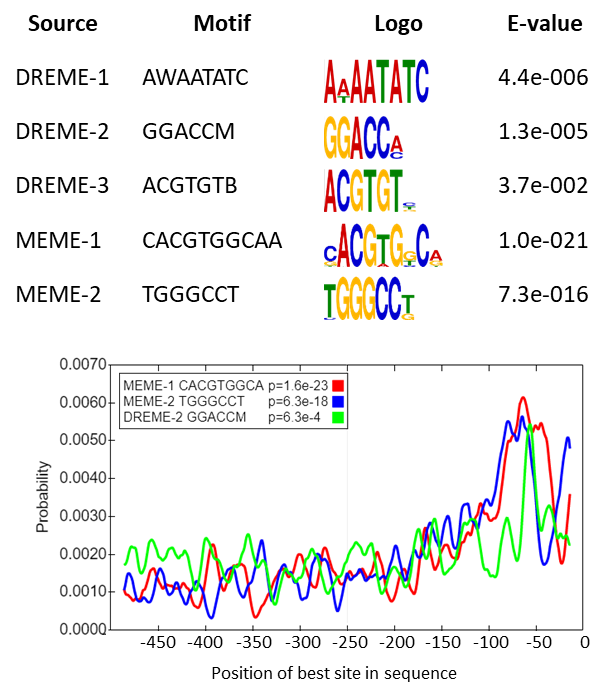
**Rhythmic White

**A**

**B**

**
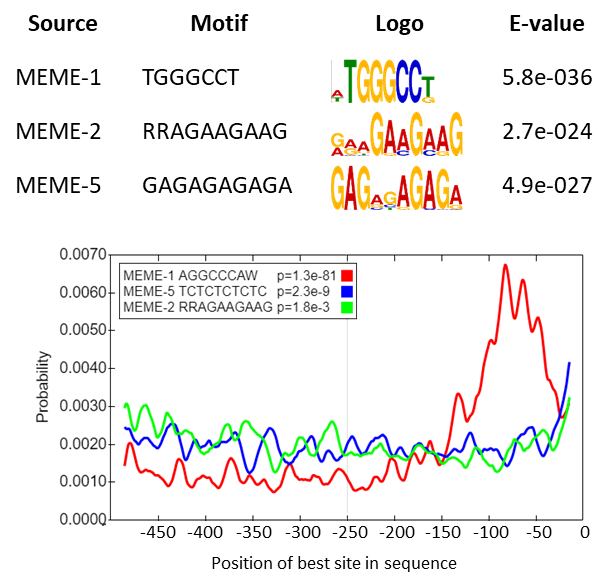
**Rhythmic Red

**
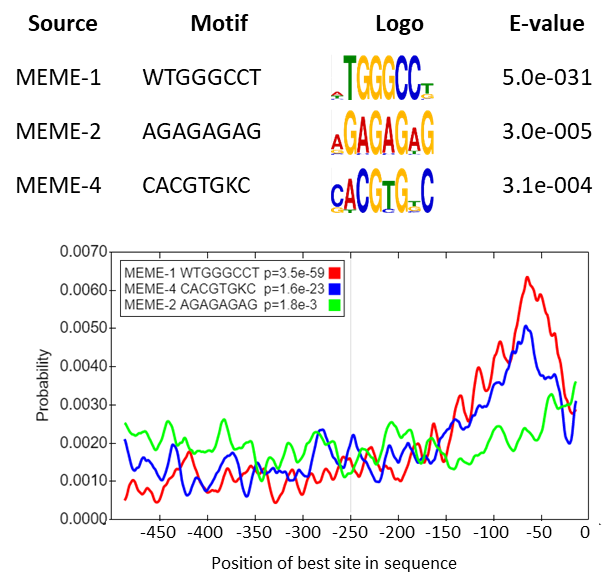
**Red -1.5x v White

**C**

**D**

**
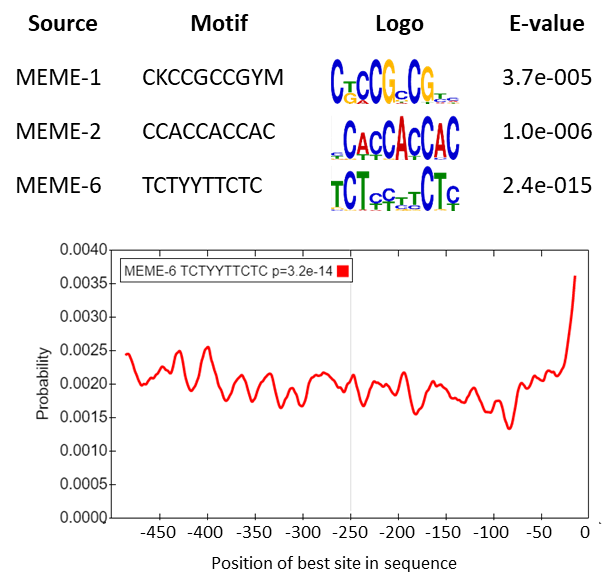
**Red +1.5x v White

**
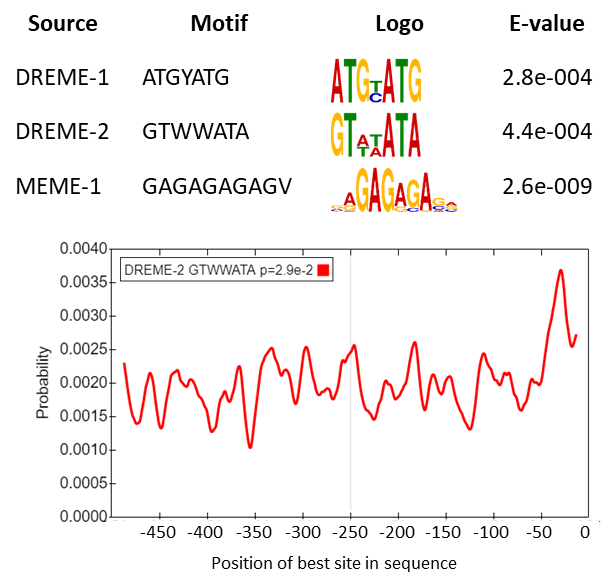
**Rhythmic *fhy3*

**E**

**F**

**
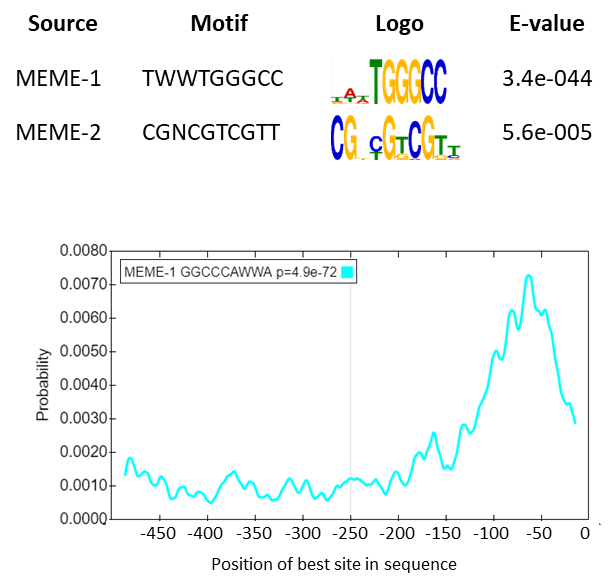
**Arrhythmic *fhy3*

**
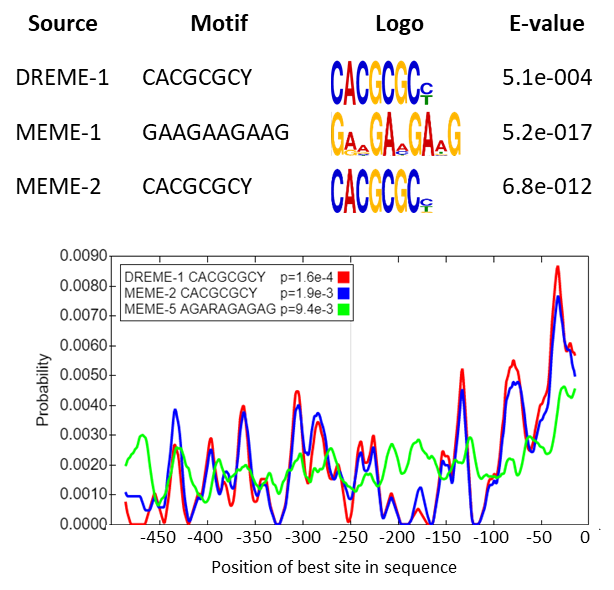
***fhy3* -1.5x v WT

**G**

**Fig. S4. Promoter elements associated with various groups of rhythmic genes show a localisation close to transcription start sites.** Overrepresented cis elements were identified within a region 500 bp upstream of the transcription start site for each group of genes using the DREME and MEME applications (Bailey and Elkan, 1994, Bailey, 2011). The groups of genes analysed and the elements identified correspond to those shown in Figure 5A. A. Rhythmic White (rhythmic in wild type in white light); B. Rhythmic Red (rhythmic in wild type in red light); C. Red -1.5x v White (rhythmic genes in white light showing mean expression in red light <= 1.5 fold that in white light); D. Red +1.5 v White (rhythmic genes in white light showing mean expression in red light >= 1.5 fold that in white light); E. Rhythmic *fhy3* (genes which maintain rhythmicity in *fhy3* in red light); F. Arhythmic *fhy3* (genes which lose rhythmicity in *fhy3* in red light); G. *fhy3* +1.5 v WT (genes with mean expression in *fhy3* in red light <= 1.5 fold that in wild type in red light). For each group of genes, the upper figure shows the elements identified and corresponding E-value associated with enrichment, while the lower figure shows any enrichment for location of each element within the 500 bp promoter sequences of genes within that group analysed using a adapted version of the Centrimo application (Bailey and Machanick, 2012).

**
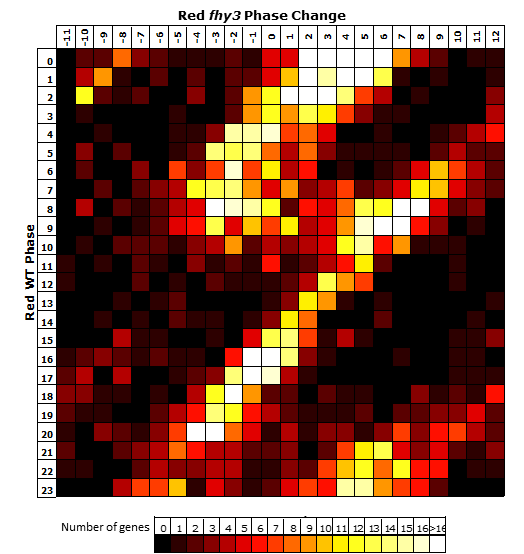
**

**Fig. S5. Genes which maintain rhythmicity in *fhy3* show a phase-dependent shift in phase.** Phase of expression in wild type seedlings in red light was plotted against shift in phase observed in the *fhy3* mutant for all genes identified as rhythmic in both wild type and *fhy3* based on microarray data. Numbers of genes displaying each phase / phase shift combination were represented as a heatmap. Phase advances are represented as negative numbers while phase delays are represented as positive.

***
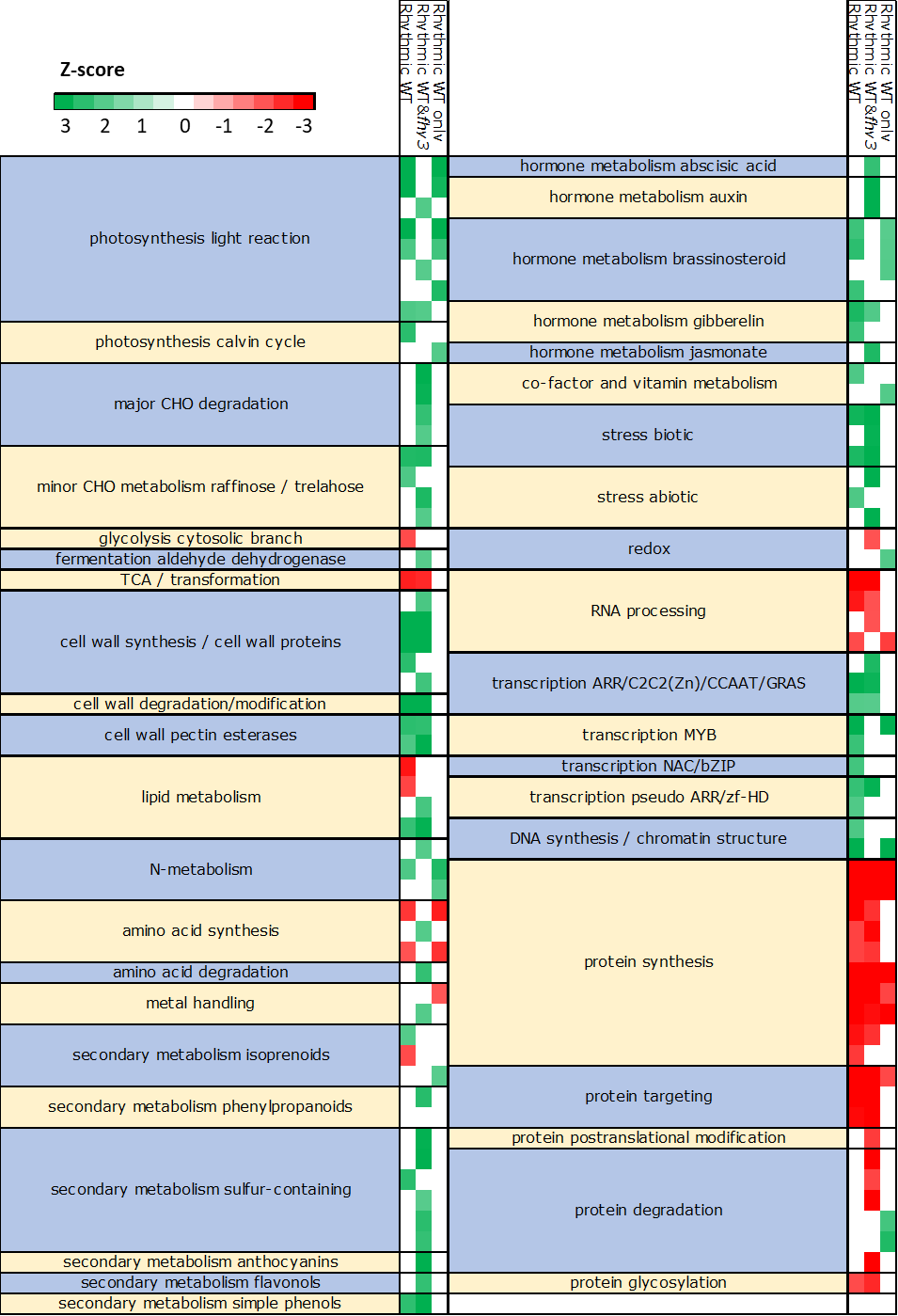
***

**Fig. S6. Rhythmicity of gene expression within specific biological processes is differentially affected by the *fhy3* mutation in red light.** Overrepresented (green) and underrepresented (red) biological process categories associated with rhythmic genes in wild type light versus the *fhy3* mutant seedlings in red light were calculated using the PageMan application. “Rhythmic WT” contains all genes rhythmic in wild type; “Rhythmic WT & *fhy3*” contains genes that were rhythmic in wild type and *fhy3*; while “Rhythmic WT only” contains genes that were rhythmic in wild type but which became arrhythmic in *fhy3*. For clarity, categories are labelled according to parent ontology terms, though enrichment patterns of all child terms are represented for each.

**Bibliography**

**Bailey, T.** (2011) DREME: motif discovery in transcription factor ChIP-seq data. *Bioinformatics*, **27**, 1653-1659.

**Bailey, T. and Machanick, P.** (2012) Inferring direct DNA binding from ChIP-seq. *Nucleic Acids Research*, **40**.

**Bailey, T.L. and Elkan, C.** (1994) Fitting a mixture model by expectation maximization to discover motifs in biopolymers. *Proc Int Conf Intell Syst Mol Biol*, **2**, 28-36.
